# Polyaspartamide-Based Nanoparticles Loaded with Fluticasone Propionate and the In Vitro Evaluation towards Cigarette Smoke Effects

**DOI:** 10.3390/nano7080222

**Published:** 2017-08-13

**Authors:** Emanuela Fabiola Craparo, Maria Ferraro, Elisabetta Pace, Maria Luisa Bondì, Gaetano Giammona, Gennara Cavallaro

**Affiliations:** 1 Laboratory of Biocompatible Polymers, Dipartimento di Scienze e Tecnologie, Biologiche, Chimiche e Farmaceutiche (STEBICEF), Università di Palermo, via Archirafi 32, 90123 Palermo, Italy; emanuela.craparo@unipa.it (E.F.C.); gaetano.giammona@unipa.it (G.G.); 2 Istituto di Biomedicina e Immunologia Molecolare, Consiglio Nazionale delle Ricerche-via Ugo La Malfa 153, 90146 Palermo, Italy; maria.ferraro@ibim.cnr.it (M.F.); elisabetta.pace@ibim.cnr.it (E.P.); 3 Istituto per lo Studio dei Materiali Nanostrutturati-U.O.S. di Palermo, Consiglio Nazionale delle Ricerche-via Ugo La Malfa 153, 90146 Palermo, Italy; marialuisa.bondi@ismn.cnr.it

**Keywords:** α,β-poly-(*N*-2-hydroxyethyl)-d,l-aspartamide (PHEA), poly(lactic acid) (PLA), poly(ethylene glycol) (PEG), *polymeric nanoparticles*, fluticasone propionate (FP)

## Abstract

This paper describes the evaluation of polymeric nanoparticles (NPs) as a potential carrier for lung administration of fluticasone propionate (FP). The chosen polymeric material to produce NPs was a copolymer based on α,β-poly(*N*-2-hydroxyethyl)-d,l-aspartamide (PHEA) whose backbone was derivatised with different molecules, such as poly(lactic acid) (PLA) and polyethylenglycol (PEG). The chosen method to produce NPs from PHEA-PLA-PEG_2000_ was the method based on high-pressure homogenization and subsequent solvent evaporation by adding Pluronic F68 during the process and trehalose before lyophilisation. Obtained colloidal FP-loaded NPs showed a slightly negative surface charge and nanometric dimensions that are maintained after storage for one year at −20 °C and 5 °C. The FP loading was about 2.9 wt % and the drug was slowly released in simulated lung fluid. Moreover, the obtained NPs, containing the drug or not, were biocompatible and did not induce cell necrosis and cell apoptosis on bronchial epithelial cells (16-HBE). Further in vitro testing on cigarette smoke extract (CSE)-stimulated 16-HBE revealed that FP-loaded NPs were able to reduce the survivin expression, while either free FP or empty NPs were not able to significantly reduce this effect.

## 1. Introduction

Fluticasone propionate (FP) is one of the most potent inhaled corticosteroids (ICS) that is commonly prescribed as the first line therapy for the asthma management [1,2,3]. FP possesses a low oral bioavailability and, for this reason, several formulations for inhalation were developed and put on the market, such as a dry powder inhalers, as a metered dose inhaler (a suspension of FP with suitable propellants in a pressurized container), and as micronized suspension for nebulization (i.e., Flixotide^®^, Aliflus^®^). As for other therapies, especially for chronic diseases, drug administration directly to the lungs, instead of oral or parenteral administrations, allows using lower doses to obtain an equivalent therapeutic response, reducing several side effects, and the toxicity of the drugs. For many of them, however, the therapeutic efficacy after inhalation is often reduced due to the fast clearance from the lung, being rapidly adsorbed and, thus, reaching the circulatory system [4]. Therefore, in order to increase the pulmonary efficacy of FP and to modulate the drug release profile, an innovative strategy could be adopted in order to prolong the FP residence time in the lung, that is, the entrapment of the drug into colloidal drug delivery systems and their administration as an aerosolized formulation [2]. Moreover, a further goal is to maximize lung residence time; mucus-penetrating particles must also be designed by realizing nanoparticles possessing a coating specifically able to minimize adhesive interactions with mucin fibers, thus penetrating readily into the pulmonary mucus layer [4,5,6]. In particular, this strategy could be targeted by the attachment of hydrophilic uncharged polymers, like polyethylenglycol (PEG) to drugs, proteins, and particles, which seems to reduce interactions with sialic acid, a major component of mucus [7,8,9]. Recently, Popov and coworkers [4] realized FP-loaded mucus-penetrating nanoparticles and, by in vivo experiments, demonstrated that the pulmonary administration achieved a higher local exposure to FP in lungs of rodents when compared to free drug, leading to a significant extension of the anti-inflammatory effect of FP in a rat lung inflammation model as compared to a non-encapsulated FP control.

In our work we described the preparation of polymeric nanoparticles (NPs) to be potentially used as drug delivery systems for pulmonary administration of FP. The applied method to obtain these particles was high-pressure homogenization (HPH), which contemplates as a final step organic solvent evaporation, while the starting material was a pegylated-polylactide graft copolymer of α,β-poly(*N*-2-hydroxyethyl)-d,l-aspartamide (PHEA), recently synthesized by our group and named PHEA-PLA-PEG_2000_ [10,11]. The suitability of the latter to realize biocompatible and either stealth or mucus-penetrating polymeric NPs was already described in the literature [12,13]. Here, the suitability of these particles to be used as carriers for pulmonary administration of FP was investigated by carrying out a proper chemical-physical and technological characterization of the obtained drug-loaded NPs. Preliminary biological tests were also carried out to evaluate biocompatibility and efficacy of FP-loaded NPs on bronchial epithelial cells (16-HBE), previously stressed by exposition to cigarette smoke extract (CSE).

## 2. Result and Discussion

### 2.1. Preparation and Characterization of Polymeric Nanoparticles (NPs) Based on PHEA-PLA-PEG_2000_ Copolymer 

In this work we described the preparation of polymeric nanoparticles (NPs) to be potentially used as drug delivery systems for fluticasone propionate (FP) in the management of lung inflammation. As the polymeric material we have chosen an amphiphilic derivative of α,β-poly(*N*-2-hydroxyethyl)-d,l-aspartamide (PHEA), previously synthesized and described in the literature, largely used as a potential carrier for biomedical applications [10,12,14,15,16]. In particular, PHEA was properly functionalized with adequate amounts of polylactide (PLA) and *O*-(2-aminoethyl)-*O*′-methylpolyethylenglycole (PEG_2000_), obtaining PHEA-PLA-PEG_2000_ graft copolymer [12].These two different functionalization reactions have a specific goal: on one hand, we chose PLA because it is an FDA-approved polymer and it allows obtaining a copolymer adequate for preparing polymeric nanoparticles because it introduces biodegradable hydrophobic chains onto PHEA [17,18,19,20]. On the other hand, we chose PEG because it is able to increase the biocompatibility of the resulting copolymer and it confers stealth and mucus-penetrating properties to the resulting polymeric carriers [7,8,9]. 

**Figure 1:**
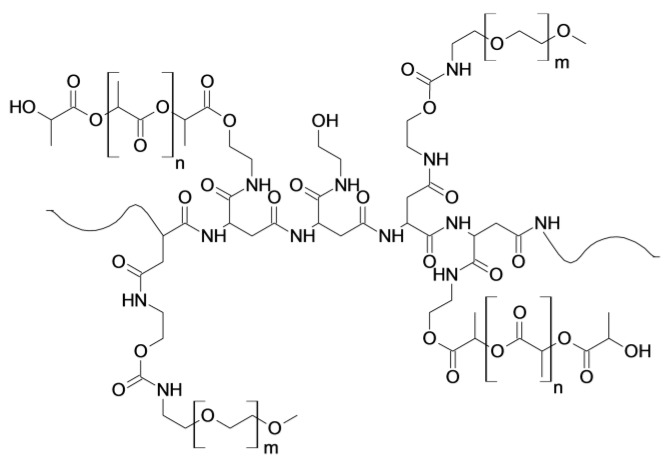
Chemical structure of PHEA-PLA-PEG_2000_ copolymer (*m* = 44, *n* = 194).

The proper characterization of obtained PHEA-PLA-PEG_2000_ copolymer by ^1^H-NMR allows determining the derivatization degree in PLA (DD_PLA_) and in PEG_2000_ (DD_PEG_) linked to the backbone, that were found to be, respectively, 4.8 mol% and 3.5 mol% with respect to the repeating units (U.R.) of PHEA. The linkage of PLA and PEG_2000_ to PHEA and PHEA-PLA copolymers, respectively, was also confirmed by SEC analysis. In particular, obtained data revealed an increase of the molecular weight of PHEA-PLA with respect to PHEA, being PHEA-PLA ${\bar{M}}_{w}$ = 93.4 kDa (with ${\bar{M}}_{w}/{\bar{M}}_{n}$ = 1.83) and PHEA ${\bar{M}}_{w}$ = 40.1 kDa; moreover, a further increase of the molecular weight of PHEA-PLA-PEG_2000_ in comparison with PHEA-PLA was detected, being PHEA-PLA-PEG_2000_ ${\bar{M}}_{w}$ = 109.0 kDa, (${\bar{M}}_{w}/{\bar{M}}_{n}$ = 1.75). It is interesting to underline that these values were close to the calculated values by considering the starting PHEA ${\bar{M}}_{w}$ and the resulting DD_PLA_ and DD_PEG_. The chemical structure of the PHEA-PLA-PEG_2000_ copolymer is schematically depicted in Figure 1.

PHEA-PLA-PEG_2000_ copolymer was used to prepare NPs by following a well-known method to produce polymeric and lipid nanoparticles, such as high-pressure homogenization (HPH), which was characterized by the use Pluronic F68 as an emulsion-stabilizing agent and a final evaporation of the organic solvent [9]. Briefly, the process involved the preparation of an oil in water (o/w) emulsion by quickly mixing the organic solution of the copolymer (containing or not the drug) with bidistilled water, which, after a proper dilution with bidistilled water, was subjected to HPH. The final process was the evaporation of organic solvent under reduced pressure. The obtained NPs were freeze-dried in the presence of trehalose, chosen as cryoprotectant.

A proper characterization of the NPs by SEC analysis permits to ascertain the absence of degradation phenomena on PHEA-PLA-PEG_2000_ copolymer, either on the backbone and/or side chains, due to the production conditions; in fact, the molecular weight value of the copolymer, obtained by dissolving a nanoparticle sample in the organic medium, was not significantly different to those obtained from the material used to realize the NPs.

To quantify the amount of FP loaded into NPs, an HPLC analysis was carried out as described in detail in the experimental part. The drug loading (DL %), expressed as weight percentage ratio between the loaded drug and the dried system (NPs + BDP + lactose), was 2.9 wt %.

It was opportune to evaluate if the obtained systems possess effectively colloidal dimensions and adequate surface properties for the aim of this work; thus, the obtained FP-loaded NPs were characterized in terms of the mean distribution size, polydispersity of the distribution, and ζ potential by using photon correlation spectroscopy (PCS) after re-dispersion in phosphate-buffered saline (PBS). Data are reported in Table 1.

It is clearly demonstrated by the reported data that our NPs show nano-scaled size and slightly negative zeta potential, probably due to the PEG chains in the starting material used to produce the nanoparticle that is preferentially exposed onto the nanoparticle surface. The more shielded surface of our NPs, thanks to the PEG presence, is confirmed by the experimental evidence that ζ potential values were significantly higher when nanoparticles were obtained by using non-pegylated copolymers, as reported elsewhere [19]. Empty NPs also showed no significant increase in mean size or alteration of zeta potential values in the same medium, before or after lyophilization (data not shown).

The evaluation of these particles to be used as a pharmaceutical formulation for pulmonary administration of FP was carried out by determining their stability after storage in terms of size, PDI, and ζ potential, according with International Conference on Harmonization (ICH) guidelines Q1A (R2) [15,21]. In particular, NPs were stored for 12 months either in a freezer at −20 °C ± 5 °C or in a refrigerator at 5 °C ± 3 °C. After this time, samples were dispersed in PBS. We firstly evaluated the physical appearance and ease of reconstitution, and then we analyzed the NPs’ dispersions in terms of mean size, PDI, and **ζ** potential. The obtained results suggested that these particles were very stable during storing in all the chosen conditions, being easily dispersed to obtain a milky aqueous dispersion. Moreover, mean size and **ζ** potential values of FP-loaded NPs were comparable to those measured in fresh dispersions, although a higher width of distribution values were found, as can be seen from data reported in Table 1. In addition, the chemical stability of FP entrapped into NPs was confirmed by HPLC analysis after storage (data not shown).

**Figure 2:**
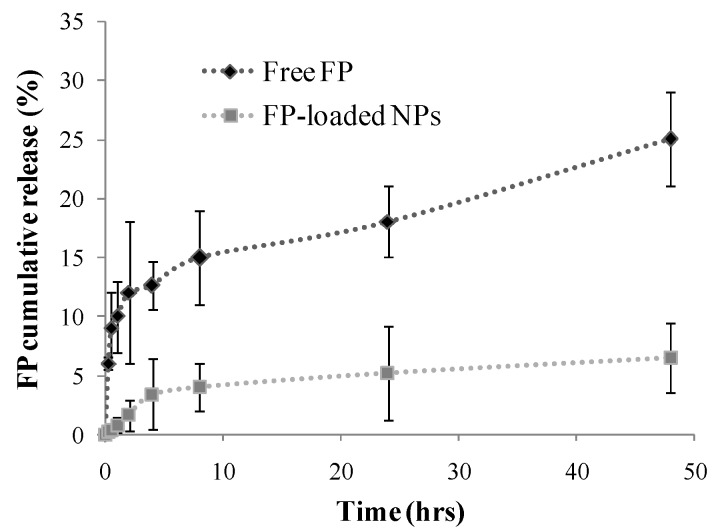
Fluticasone propionate (FP) diffusion profile and release profile of FP from PHEA-PLA-PEG_2000_ NPs in SLF at pH 7.4. Data represent mean ± S.D. (*n* = 3).

To evaluate the ability of these NPs to act as a drug delivery system for FP, thus to slowly release the encapsulated drug under sink conditions in physiological media, a drug release study was carried out in simulated lung fluid (SLF) at pH 7.4 by evaluating the amount of released drug from NPs at prefixed time intervals across a dialysis tube. Moreover, the FP diffusion profile alone was investigated in order to determine the diffusion rate of the free drug across the dialysis membrane. The amount of released FP was expressed as the percentage ratio between the weight of the cumulatively-released drug at the prefixed time and the total amount of FP loaded into NPs. In Figure 2, the drug dissolution and release profile from PHEA-PLA-PEG_2000_ NPs were reported until 48 h incubation.

Results clearly indicate that the investigated carriers are able to retain more than 90 wt % of initially-entrapped drug even after 48 h incubation; this behavior could be positively exploited to improve the drug internalization into cells being drug loaded nanoparticles potentially able to enter into the cells and, once inside, could release the drug.

Considering results concerning chemical-physical characterization and stability on storage, these FP-loaded NPs could be potentially administered by nebulization of a nano-scaled suspension; the latter could be extemporaneously obtained by dispersing the lyophilized system in the physiological isotonic aqueous solution. In this case, it is the size of the aqueous droplet that determines the fate of the inhaled nanocarriers [1].

### 2.2. Biological Characterization

**Figure 3:**
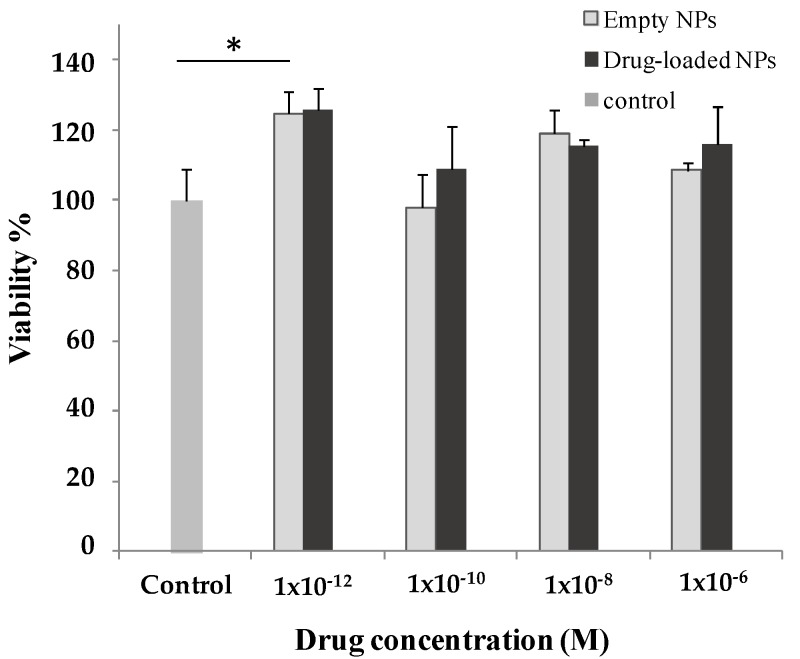
Effects of empty and FP-loaded PHEA-PLA-PEG_2000_ NPs (corresponding to drug concentrations ranging between 10^−^^6^ and 10^−^^12^ M) on 16-HBE cell viability. Amounts of empty NPs were equal to those used for drug-loaded NPs. Data represent mean ± S.D. (*n* = 3). * *p* < 0.05.

In order to evaluate the biocompatibility of empty and FP-loaded PHEA-PLA-PEG_2000_ NPs, in vitro assays were performed on human bronchial epithelial cell line (16-HBE). In particular, the metabolic activity was evaluated by MTS viability assay. Data, reported in Figure 3, demonstrated that incubation of 16-HBE in the presence of FP-loaded NPs (corresponding at drug concentrations ranging between 10^−6^ and 10^−12^ M of drug) did not modify cell metabolic activity. The same results were obtained in the presence of empty NPs, incubated with cells at concentrations equal to those used for drug-loaded NPs. 

**Figure 4:**
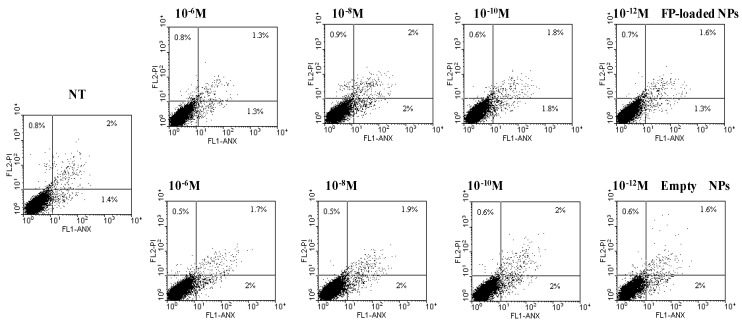
Effects of empty and FP-loaded NPs on cell necrosis and cell apoptosis of 16-HBE by using Annexin V/Propidium Iodide method by flow cytometry. Representative dot plots out from three different experiments were shown. The propidium-negative and annexin V-negative cells (i.e., viable cells) are present in the lower left quadrant; the propidium-positive cells (i.e., necrotic cells) are present in the upper left quadrant; the propidium and annexin V double-positive cells (i.e., late apoptotic cells) are present in the upper right quadrant and the single annexin V-positive cells (i.e., early apoptotic cells) are present in the lower right quadrant.

Furthermore, cell necrosis/cell apoptosis were evaluated by annexin V/propidium iodide method [22,23]. As shown in representative dot plots of Figure 4, neither empty, nor FP-loaded, NPs (corresponding to drug concentrations ranging between 10^−6^ and 10^−12^ M) induced cell apoptosis or necrosis. All these data support the belief that FP-loaded PHEA-PLA-PEG_2000_ NPs are well tolerated by bronchial epithelial cells. 

### 2.3. Biological Efficacy

The airway epithelium shows active defense mechanisms by releasing cytoprotective mucus and defensins, and exerts an important role in coordinating local inflammation and innate immune responses [24]. Environmental stress, including cigarette smoke, hastens the shriveling of the tips of telomeres and alters the phenotype and the metabolism, thus shortening cellular life span and accelerating the process of premature senescence [25]. When epithelial cells are chronically exposed to cigarette smoke, they differentiate in a functional senescent phenotype with a reduced reparative potential and higher pro-inflammatory properties. Survivin is essential in protecting cells from entering apoptosis and in controlling cell growth.

Here, to test whether FP loaded into PHEA-PLA-PEG_2000_ NPs was more effective than free FP, we assessed the expression of survivin, a protein that contributes to the apoptosis resistance observed in aged, as well as in senescent cells [24]. Cigarette smoke extract (CSE) or an inhibitor of the deacetylase activity of SIRT1 (sirtinol), increased the expression of survivin protein and this CSE mediated effect was associated to reduced localization of FoxO_3_ on the survivin gene promoter in bronchial epithelial cells [26].

**Figure 5:**
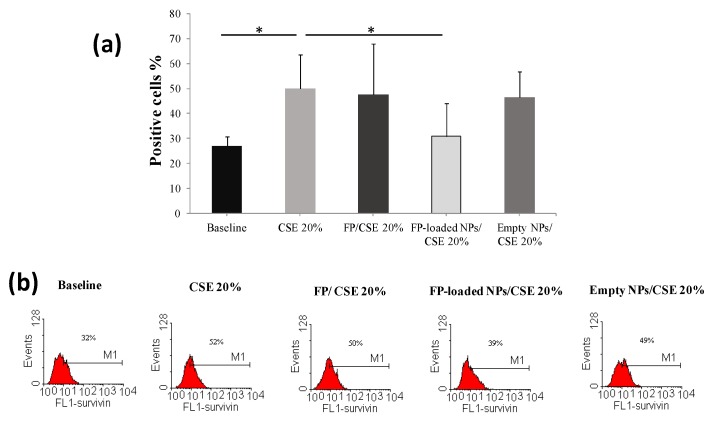
Effects of free FP, FP-loaded NPs, and empty NPs on survivin expression in CSE-exposed cells. Cells were exposed to CSE 20% in the presence and in the absence of free FP, FP-loaded PHEA-PLA-PEG_2000_ NPs, and empty NPs, and survivin expression was assessed by flow cytometry. Data were expressed as the percentage of positive cells (mean ± S.D.) (*n* = 5) (**a**). Representative histograms are shown in (**b**) (Fluorescence intensity –FL1). * *p* < 0.05.

**Figure 6:**
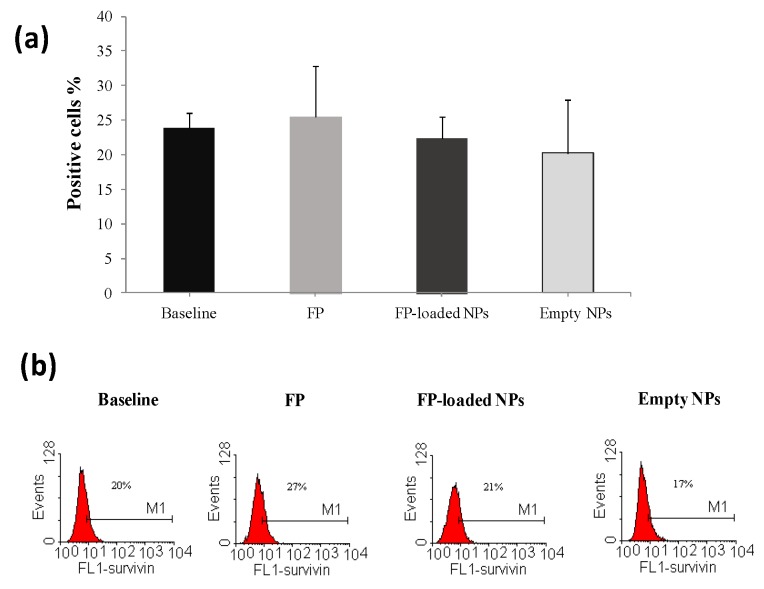
Effects of unloaded FP, FP-loaded, and empty NPs on survivin expression in 16-HBE. Cells were cultured in the presence and in the absence of free FP or FP-loaded PHEA-PLA-PEG_2000_ NPs and and survivin expression was assessed by flow cytometry. Data were expressed as the percentage of positive cells (means ± S.D.) (*n* = 3) (**a**). Representative histograms are shown in (**b**) (Fluorescence intensity –FL1).

In Figure 5 and Figure 6, effects on survivin expression of FP, free and loaded into PHEA-PLA-PEG_2000_ NPs, respectively, in CSE-exposed or untreated cells, are reported, at a drug concentration of 10^−8^ M. Moreover, effects on survivin expression in the presence of empty NPs was evaluated, at a concentration equal to that used for drug-loaded NPs. 

Obtained data demonstrated that free FP or FP-loaded PHEA-PLA-PEG_2000_ NPs did not significantly modify the constitutive expression of survivin in 16-HBE (Figure 6), while the incubation in the presence of FP-loaded PHEA-PLA-PEG_2000_ NPs significantly reduced survivin expression in cells exposed to CSE (with increased survivin expression) (Figure 5). Indeed, the capability to counteract this CSE-mediated effect was not found for free FP. This effect on survivin expression could be explained considering the enhancement of FP uptake and internalization in 16-HBE exposed to CSE when it is entrapped into our polymeric carriers, that interact with cell membrane and could promote the uptake of encapsulated drug via NPs endocytosis and/or drug diffusion [9,13].

**Figure 7:**
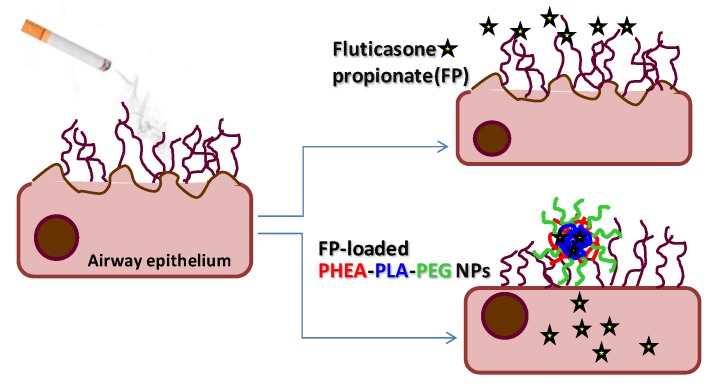
Schematic representation of potential effects of FP-loaded NPs strategy after exposition of airway epithelium to cigarette smoke.

In conclusion, the present study provides evidences that these NPs may be considered a promising strategy to counteract cigarette smoke-induced events not controlled by corticosteroids, as schematically depicted in Figure 7.

## 3. Materials and Methods 

### 3.1. General Methods 

Anhydrous *N*,*N*′-dimethylformamide (a-DMF), anhydrous dimethylacetamide (a-DMA), 1,1′-carbonyldiimidazole (CDI), d,l-poly(lactic acid) (PLA acid terminated, 10–18 kDa), poly(ethylene oxide) standards and fluticasone propionate (FP), were purchased from Sigma-Aldrich (Milan, Italy). Triethylamine (TEA), diethylamine (DEA), ethyl ether, *O*-(2-Aminoethyl)-*O*′-methyl poly(ethylene glycol) 2000 (H_2_N-PEG_2000_) (≤0.4 mmol NH_2_/g), Pluronic F68 and dichloromethane were obtained from Fluka (Milan, Italy). All used reagents were of analytic grade.

α,β-Poly(*N*-2-hydroxyethyl)-d,l-aspartamide (PHEA) was obtained and characterized as reported in the literature [10]. ^1^H-NMR (300 MHz, D_2_O, TMS): δ 2.8 (m, 2H, -CH-CH_2_-C(O)NH-), δ 3.2 (m, 2H, -NH-CH2-CH2-OH), δ 3.5 (m, 2H, -NH-CH_2_-CH_2_-OH), δ 4.6 (m, 1H, -NH-CH(CO)CH_2_-). Size exclusion chromatography (SEC) was used to evaluated the weight average molecular weight (${\bar{M}}_{w}$) of PHEA, that resulted to be 40.1 kDa (${\bar{M}}_{w}/{\bar{M}}_{n}$ = 1.58). 

SEC analysis was carried out by using as an eluent 0.01 M LiBr DMF solution and a flow of 0.8 mL/min at 50 °C, by constructing a curve of calibration with poly(ethylene glycol) standards (range 145–1.5 kDa) and by using a Waters instrument (Waters, Mildford, MA, USA) and two columns from Phenomenex with 5 μm particle size (103 Å and 104 Å of pores size, Phenogel, Milan, Italy), and a 410 differential refractometer.

### 3.2. Synthetic Procedures and Nanoparticle Formation

#### 3.2.1. Synthesis of PHEA-PLA Graft Copolymer and Characterization

The chemical functionalization of PHEA with chains of acid terminated PLA was done by an already reported method, obtaining the PHEA-PLA graft copolymer with a yield of 310 wt % respect to the starting PHEA [19]. ^1^H-NMR (300 MHz, DMF-d7, 25 °C, TMS): δ 1.3 and δ 1.7 (2d, 582 H_PLA_ -O-CO-CH(CH_3_)-O-); δ 2.8 (m, 2H_PHEA_ -CO-CH-CH_2_-CO-NH-); δ 3.3 (t, 2H_PHEA_ -NH-CH_2_-CH_2_-O-); δ 3.6 (t, 2H_PHEA_ -NH-CH_2_-CH_2_-O-); δ 4.2–4.5 and δ 5.1–5.5 (m, 194 H_PLA_ -O-CO-CH(CH_3_)-), and δ 4.8 (m, 1H_PHEA_ -NH-CH(CO)CH_2_-).

#### 3.2.2. Synthesis of PHEA-PLA-PEG_2000_ Graft Copolymer and Characterization

The chemical reaction of PHEA-PLA with chains of PEG_2000_ was done following an already reported method, obtaining the PHEA-PLA-PEG_2000_ with a yield of 98.6 wt % respect to PHEA-PLA [9].^1^H-NMR (300 MHz, DMF-d7, 25 °C, TMS): δ 1.5 and δ 1.9 (2d, 582 H_PLA_ -O-CO-CH(CH_3_)-O-); δ 2.9 (m, 2H_PHEA_ -CO-CH-CH_2_-CO-NH-); δ 3.5 (t, 2H_PHEA_ -NH-CH_2_-CH_2_-O-); δ 3.7 (t, 2H_PHEA_ -NH-CH_2_-CH_2_-O-); δ 3.8 (t, 176 H_PEG_ -CH_2_-CH_2_-O-); δ 4.3–4.6 and δ 5.3–5.5 (m, 194 H_PLA_ -O-CO-CH(CH_3_)-), and δ 5.0 (m, 1H_PHEA_ -NH-CH(CO)CH_2_-).

#### 3.2.3. General Procedure for Nanoparticle Preparation 

High-pressure homogenization (HPH) was used as procedure to obtain PHEA-PLA-PEG_2000_ based nanoparticles (NPs) [13]. Based on this method, a PHEA-PLA-PEG_2000_ dispersion in dichloromethane at a concentration of 16.7 mg/mL (6 mL) was used as organic phase and mixed by stirring at 20,500 rpm with an aqueous phase (50 mL) containing Pluronic F68 (15 mg). After addition of bidistilled water (25 mL), the oil in water (o/w) emulsion was homogenized four times at 7500 psi by using an EmulsiFlexTM-C5 as a homogenizer (Avestin Inc., Ottawa, ON, Canada). Evaporation of organic solvent under reduced pressure by using a evaporation system constituted by a water bath B-480, a rotavapor R-114, an F-105 recirculating chiller and a V-800 vacuum controller (Buchi) allows obtaining empty NPs. FP-loaded NPs were prepared by dissolving the drug in the organic phase at a copolymer/drug weight ratio of 100:8.7 before starting, and then following the procedure described above. Once obtained, the NP dispersion was centrifuged at 8000 rpm for 10 min. Finally, each NP batch was dried by using a Modulyo freeze-dryer (Labconco Corporation, Kansas City, MO 64132, USA) after the addition of trehalose as a cryoprotectant at a nanoparticle/trehalose weight ratio equal to 1:1, and stored at −20 °C for successive characterization. 

### 3.3. NPs Characterization

#### 3.3.1. Mean Dimensions and ζ Potential 

The mean number distribution diameter of NPs were performed by photon correlation spectroscopy (PCS) by using the Zetasizer Nano ZSP (Malvern Instrument, Rome, Italy). Analyses were done at a fixed angle of 173° and at 25 °C by dispersing each sample in phosphate buffer saline (PBS) medium. ζ potential measurements were conducted by dispersing each sample in PBS and by analyzing it with the Zetasizer Nano ZSP.

#### 3.3.2. Drug Loading 

The amount of FP entrapped into NPs was determined by HPLC, by using a C18 column (Luna C18, 5 μm, 250 × 46 mm i.d., Phenomenex), a mixture acetonitrile:water 60:40 *v*/*v* as the mobile phase and a flow rate of 1 mL·min^−1^, detecting the drug at λ 239 nm. A proper amount of NPs (5 mg) was dispersed in acetonitrile (4 mL), filtered with Nylon filters (0.2 μm) and analyzed. DL%, expressed as the weight percent ratio between entrapped FP and the dried system (NPs + drug+ trehalose), was determined by comparing the obtained peak area corresponding to FP extracted from NPs with a calibration curve obtained by plotting areas versus standard solution concentrations of FP in acetonitrile in the range of 100–25 μg·mL^−1^ (y = 10209 × 103x, R_2_ = 0.999). 

#### 3.3.3. Stability Studies

Physical and chemical stability of FP-loaded NPs was investigated by following the guidelines of the International Conference on Harmonization (ICH) Q1A (R2) [21]. In detail, lyophilized samples were stored for 12 months in the dark, either in a freezer at −20 °C ± 5 °C or in a refrigerator at 5 °C ± 3 °C. After redispersion in PBS, physical appearance and ease of reconstitution were evaluated. In addition, mean size and ζ potential in PBS were analyzed, while chemical stability of the entrapped drug was determined by HPLC analysis, by following the method reported above.

#### 3.3.4. Drug Release in Simulated Lung Fluid (SLF)

The dialysis method was chosen to evaluate the FP release from drug-loaded NPs in sink conditions by dispersing NPs in SLF, which was prepared accordingly with reported composition [27]. To determine the drug release profile, FP-loaded NPs (0.5 mg) were dispersed in the medium (5 mL), placed in a dialysis bag [molecular weight cut off (MWCO) 12–14 kDa and immersed in the same medium (45 mL), incubating the system in a thermostatic shaker (100 rpm, 37 °C). At prefixed time points, the receiver medium was taken, replaced by fresh medium and dried by lyophilization. The cumulative FP release percentage was determined by the HPLC method described above, after addition of acetonitrile. Data were correct taking in account the dilution procedure. In parallel, FP diffusion profile was evaluated by dispersing a proper drug amount in SLF, put the obtained dispersion in the dialysis tube (MWCO 12–14 kDa) and following the procedure reported above. 

### 3.4. Biological Characterization 

#### 3.4.1. Cell Culture 

In this study, an immortalized normal bronchial epithelial cell line (16-HBE) was used [28]. 16-HBE is a cell line that retains the differentiated morphology and function of normal airway epithelial cells. The cells represent a clonal diploid (2*n* = 6) cell line isolated from human lung previously used to study the functional properties of bronchial epithelial cells in inflammation and repair processes. 16-HBE cells were maintained in a humidified atmosphere of 5% CO_2_ in air at 37 °C, cultured as adherent monolayers in Eagle’s minimum essential medium (MEM) (Gibco, BRL, Berlin, Germany), supplemented with 10% heat-inactivated (56 °C, 30 min) fetal bovine serum (FBS) (Gibco), 1% MEM (non-essential aminoacids) (Euroclone), 2 mM *L*-glutamine, and 0.5% gentamicin (Gibco). 

#### 3.4.2. Cell Viability Assay

Cell viability was evaluated by CellTiter 96 Aqueous One Solution Cell Proliferation Assay (PROMEGA, Madison WI USA), which contains MTS (3-(4,5-dimethylthiazol-2-yl)-5-(3-carboxymethox-yphenyl)-2-(4-sulfopheyl)2H-tetrazolium), according to the manufacturer’s instructions. In particular, cells were plated in 96-well plate and then treated for 24 h with FP-loaded NPs (at drug concentrations ranging between 10^−6^ and 10^−12^ M). Moreover, cell viability was also carried out by incubating cells in the presence of empty NPs, at concentrations equal to those used for drug-loaded NPs. After incubation time, 20 mL of One Solution reagent was added to each well, and incubated for 20 min for the 16-HBE at 37 °C, 5% CO_2_. The absorbance was read at 490 nm on a wallacVictor2 1420 Multilabel Counter (Perkin Elmer, Milan, Italy). Results are expressed as absorbance values.

#### 3.4.3. Cell Apoptosis 

Cell apoptosis in the presence of FP-loaded NPs (at drug concentrations ranging between 10^−6^ and 10^−12^ M) was evaluated by staining with annexin V-fluorescein isothiocyanate and propidiumiodide (PI) using a commercial kit (Bender Med-System, Vienna, Austria) following the manufacturer’s directions. Moreover, cell apoptosis was also carried out in the presence of empty NPs, at concentrations equal to those used for drug-loaded NPs. Cells were analyzed using a FACSCalibur (Becton Dickinson, Mountain View, CA, USA) analyzer equipped with an argon ion laser (Innova 70 Coherent, Santa Clara, CA, USA) and Consort 32 computer support.

#### 3.4.4. Preparation of Cigarette Smoke Extract (CSE)

Cigarette smoke solution was prepared from commercial cigarettes) as described previously [29]. Each cigarette was smoked for 5 min, and two cigarettes were used per 20 mL of PBS to generate a CSE-PBS solution. The CSE solution was filtered through a 0.22 μm pore filter to remove bacteria and large particles. The smoke solution was then adjusted to pH 7.4 and used within 30 min of preparation. This solution was considered to be 100% CSE and diluted to obtain the desired concentration for each experiment. The concentration of CSE was calculated spectrophotometrically, measuring the optical density (OD) as previously described [29] at the wavelength of 320 nm. The pattern of absorbance, among different batches, showed very few differences, and the mean OD of the different batches was 1.37 ± 0.16. The presence of contaminating LPS on undiluted CSE was assessed by a commercially available kit (Cambrex Corporation, East Rutherfort, NJ, USA) and was below the detection limit of 0.1 EU/mL.

#### 3.4.5. Stimulation of 16-HBE

Cells were grown in polystyrene flasks 25 cm^2^ (BD Falcon, Franklin Lakes, NJ, USA) for three days until 80–90% confluency. Then, 16-HBE cells were treated in 1% FBS with CSE (20%) medium, and FP, FP-loaded NPs (both at a FP concentration equal to 10^−8^ M), and empty NPs at a concentration equal to that used for drug-loaded NPs, were added 1 h before CSE cell stimulation. The time of incubation of CSE is 24 h to assess the biocompatibility and survivin expression. The concentration of FP and FP-loaded NPs was selected on the basis of preliminary experiments. The concentration of CSE and the time of incubation were selected on the basis of previous findings [22]. At the end of stimulation, cells were collected for further evaluations. At least three replicates were performed for each experiment.

#### 3.4.6. Survivin Expression

The expression of survivin was evaluated by flow-cytometry. Flow cytometry analyses were performed on a FACSCalibur (Becton Dickinson, Mountain View, CA, USA). The cells (16-HBE) were washed twice in PBS and fixed with PBS containing 4% paraformaldehyde for 20 min at room temperature. After two washes in permeabilization buffer (PBS containing 1% FCS, 0.3% saponin, and 0.1% sodium azide) for 5 min at 4 °C cells were incubated with an anti-rabbit Survivin (Novus Biologicals, Littleton, CO, USA), followed by anti-rabbit IgG FITC (1:350) (Antibodies online) IgG. Analysis was conducted on 10,000 acquired events for each sample using cellQuest acquisition and data analysis software (Becton Dickinson, San Jose, CA 95131-1807, USA). Negative controls were performed using an isotype control antibody (BD PharMingen, Mountain View, CA, USA). Data are expressed as the percentage of positive cells.

### 3.5. Statistical Analysis

All the experiments were conducted in triplicate, expressing data as means ± standard deviation (S.D.) and analyzing them via Student’s *t*-test. A *p*-value < 0.05 was considered as statistically significant, while a *p*-value < 0.01 was considered as highly significant.

## 4. Conclusions

In this work, we described a simple method to produce of polymeric nanoparticles (NPs) containing fluticasone propionate (FP), and their characterization in order to evaluate their potential use in improving the management of lung diseases by local application of corticosteroids. To do this, FP-loaded NPs based on an amphiphilic polyaspartamide derivative, the PHEA-PLA-PEG_2000_ graft copolymer, were successfully prepared by using the high-pressure homogenization-solvent evaporation method. In particular, physical-chemical characteristics, such as nanometric dimensions and low ζ potential values, pegylation, and stability in storage, indicate that these NPs are adequate for lung administration, potentially able to reach all lung compartments and improving drug permeation through the mucus layer. Biological assays on 16-HBE evidenced the high biocompatibility of the obtained NPs and the relevant efficacy in reducing the increased survivin expression in these cells due to cigarette smoke exposure. 

## Figures and Tables

**Table 1:** Mean size, PDI, and ζ potential values in phosphate-buffered saline (PBS) of FP-loaded NPs freshly dispersed (t_0_), after freeze-drying, upon storage for 12 months at −20 °C (t_12months_, −20 °C) or at 5 °C (t_12months_, 5 °C).

**Mean Size (nm) (±S.D.)**			
t_0_	After freeze-drying	t_12months_, −20 °C	t_12months_, 5 °C
147.4 ± 11.0	161.3 ± 14.0	204.7 ± 34.7	198.9 ± 22.6
**ζ Potential (mV) (±S.D.)**			
t_0_	After freeze-drying	t_12months_, −20 °C	t_12months_, 5 °C
−6.9 ± 1.5	−4.6 ± 2.3	−3.8 ± 3.5	−4.5 ± 3.7
